# High Definition Liposuction Classification

**DOI:** 10.1097/GOX.0000000000002440

**Published:** 2019-09-30

**Authors:** Javier Vera Cucchiaro

**Affiliations:** From the Aesthetic and Laser Surgery Clinic, Salta, Argentina.

## Sir,

In our High Definition Liposuction routine, we do not use fatty grafts at the level of the rectus abdominis and we use deep liposuction of the entire abdomen to later mark the semilunar lines, linea alba, iliac crest, and horizontal lines of the rectus.

In our practice, >50% of patients have already undergone previous lipoaspirations. In cases without previous liposuction, we use the ultrasound assisted liposuction (UAL), and in secondary cases, the laser assisted liposuction (LAL), because the chromophore is the water and with a good tumescent infiltration we can obtain less bleeding. In selected cases and without a history of thrombosis, we use the tranexamic acid diluted in the infiltration, one ampoule per liter up to a total of 3 ampoules per patient. We normally use 4–6 L of tumescent infiltration with lidocaine and under moderate sedation monitored by anesthesiologist, with a 4-hour outpatient hospital stay.

For this reason, we can make a classification of 3 types of muscle definition: soft, intermediate, and high. The soft definition is for female patients and consists of marking the semilunar lines and the line alba at the supraumbilical level (it does not exist at the infraumbilical level). The intermediate definition is for men who are not very athletic or athletic women and consists of marking, in addition to the mentioned areas, the iliac crest and its inferior portion, giving a “V” appearance to the lower abdomen. The high definition is for athletic men and exceptionally athletic women (it gives a very masculine look), and the serratus and metamers of the rectus abdominis will be marked. We tried not to make incisions at the supraumbilical level and with long, curved cannula being able to access from umbilicus and a submammary access in the women and areolar in the man.

In cases of skin flaccidity, it is combined with a cutaneous resection and transverse plication type TULUA at the infraumbilical level. TULUA is the initials of the technique described by Villegas and means: Transverse plication hypogastrium, Umbilicoplasty skin graft, Liposuction full, Undermining not up to the umbilicus and Abdominoplasty.

In women, it is associated with gluteal filling at the subcutaneous level and lipoaspiration of neighboring areas and mammary surgery. The gluteal augmentation helps to give more contrast to the region of the flanks. In men, according to the amount of fat removed, the areas to be filled are coordinated before surgery, being more frequent at the gluteal, pectoral, and shoulder levels. It is very common to treat gynecomastia with lipoaspiration, resection of mammary gland by transmammilar approach, and placement of intramuscular fat in the pectoral in the upper half of said area. The filling of fat in the deltoids and biceps is reabsorbed up to 70% because they are very mobile areas, unlike the buttocks and pectorals that only reabsorb 30%.

It can also be associated with lipoabdominoplasty using progressive traction sutures and high-definition sutures.

